# Targeting the MALAT1 gene with the CRISPR/Cas9 technique in prostate cancer

**DOI:** 10.1186/s41021-022-00252-3

**Published:** 2022-09-26

**Authors:** Soraya Ahmadi-Balootaki, Abbas Doosti, Mojtaba Jafarinia, Hamed Reza Goodarzi

**Affiliations:** 1 Department of Genetic, Marvdasht Branch, Islamic Azad University, Marvdasht, Iran; 2 Biotechnology Research Center, Shahrekord Branch, Islamic Azad University, Shahrekord, Iran; 3 Department of Biology, Marvdasht Branch, Islamic Azad University, Marvdasht, Iran; 4 Department of Genetic, Marvdasht Branch, Islamic Azad University, Marvdasht, Iran

**Keywords:** Prostate cancer, MALAT1, Knockout CRISPR/Cas9

## Abstract

**Background:**

The MALAT1 lncRNA acts as an oncogene in Prostate cancer (PC); thus, it can be severe as a cancer biomarker.

**Methods:**

Using bioinformatics datasets including (HTSeq-Counts, GDC, and TCGA) 5501 gene expression profiling specimens were gathered. Then, expression profiles and sample survival of lncRNA were investigated using COX regression analyses, ROC curve analysis. The Database for Annotation, Visualization, and Integrated Discovery was used to conduct GO and KEGG studies on the lncRNA-related PCGs. After MALAT1 Knockout via CRISPR/Cas9 technique, the MALAT1 expression was assessed in DU-145 cells. The deletion of the target fragment was examined by polymerase chain reaction (PCR). Also, the expression of apoptosis genes was investigated by qRT-PCR. The viability and cell proliferation were measured using the MTT assay. Cell migration capability was determined using the cell scratch assay. The results of qRT-PCR were assessed by the ΔΔCt method, and finally, statistical analysis was performed in SPSS software.

**Results:**

A maximum of 451 lncRNAs were discovered to reflect different expressions between PC and non-carcinoma tissue samples, with 307 being upregulated and 144 being down-regulated. Thirty-six lncRNAs related to OS were carefully selected, which were then subjected to stepwise multivariate Cox regression analysis, with 2 lncRNAs (MALAT1, HOXB-AS3). MALAT1 is highly expressed in PC cells. MALAT1 Knockout in DU-145 cells increases apoptosis and prevents proliferation and migration, and DU-145 transfected cells were unable to migrate based on the scratch recovery test. Overall, data suggest that MALAT1 overexpression in PC helps metastasis and tumorigenesis. Also, MALAT1 knockout can be considered a therapeutic and diagnostic target in PC.

**Conclusion:**

Targeting MALAT1 by CRISPR/Cas9 technique inhibit the cell proliferation and migration, and in addition induce apoptosis. Thus, MALAT1 can act as a tumor biomarker and therapeutic target.

**Supplementary Information:**

The online version contains supplementary material available at 10.1186/s41021-022-00252-3.

## Introduction

Cancer is among the diseases with a high mortality rate worldwide [1]. Its prevalence is variable in various geographic regions [2]. In developing countries, e.g., Iran, cancer is the third cause of death following cardiovascular and motor vehicle accidents [3–5]. In Iran, cancer risk is higher among males than females; moreover, the cancer incidence before 75 years is approximately 13.1% [2, 6]. Prostate cancer (PC) is one of the most prevalent cancers among males globally [3]. Also, following lung cancer, PC is the second cause of mortality in males [7]. Compared with Western countries, its incidence in Asian countries is lower [2, 8, 9], e.g., this cancer is the eighth cause of mortality in Iran [10, 11]. PC is age-dependent [12], and Adenocarcinoma is the most prevalent PC, more common in patients over the age of 65 years [9]. Prostate cancer accounts for 7-9% of total cancers among Iranian males [13]. This cancer has enhanced among hormone-dependent cancers in the past decade by approximately about 11% [10, 11]. The definite cause of PC is unknown, though environmental and genetic factors are involved [14]. According to previous studies, only 2% of the human genome comprises protein-coding genes [15], while the remaining 98% are non-coding [16]. These non-coding genes were first considered junk DNA [17] and without biological function [18]. However, they were not defined as transcriptional noise [19] since it was found that the majority of these DNA parts encode functional non-coding RNAs (ncRNAs) [20]. ncRNAs are a large group of RNA molecules that act as housekeeping and regulatory RNAs. Regulatory RNAs are divided into two groups based on their size: 1) small non-coding RNAs with a length of less than 200 bp [21, 22], 2) long non-coding RNAs (lncRNAs) ranging from 200 nucleotides to more than 50 kilobases [23]. Most ncRNA transcripts are composed of lncRNAs [21]; however, the function of many of them is still unknown [23]. Studies have shown that the human genome has more lncRNA genes than protein-coding genes [24]. Many lncRNAs, such as protein-coding genes, are transcribed by RNA polymerase II [25]; both carry genetic information [26] and can be capped and polyadenylated [27]. lncRNAs are usually regulated utilizing transcriptional factors and are expressed specifically in the tissue [25]. However, they can function very differently. Based on their position in the genome, lncRNAs are classified into four categories: 1) intronic lncRNAs; 2) intergenic lncRNAs; 3) sense and antisense lncRNAs, and 4) the last group is not placed in other groups due to structural complexity. The processed transcripts are located in a locus and have no open reading frame (ORF) [26]. lncRNAs interact with proteins, RNA, and DNA and are involved in gene regulation [28]. lncRNA expression in cancer is the product of abnormal gene expression. Moreover, genetic mutations can affect lncRNA expression [29]. Therefore, lncRNAs are the main regulators of gene expression via processes such as transcriptional and post-transcriptional, chromatin modification, genomic imprinting, and mRNA splicing [17, 30, 31]. Despite not being protein-coding, lncRNAs play a vital role in cellular functions [32]. Metastasis-associated lung adenocarcinoma transcript 1 (MALAT1) is one of the lncRNAs associated with malignancy [33]. This lncRNA with a length of 8.5 kb is located in chromosome 11q13 [34]. MALAT1 was named based on its clinical importance in metastasis anticipation, its high expression in metastatic samples, and survival in early-phase non-small cell lung carcinoma (NSCLC) [34–41]. According to studies, MALAT1 was identified as one of the primary lncRNAs, in cancer and a cancer biomarker. MALAT1 upregulation is known in different cancers, including prostate, colorectal, esophageal, endometrial, melanoma, hepatocellular, and ovarian cancers. MALAT1 operates as an oncogene in multiple cancers characterized by extremely elevated expression [42]. Despite the role of MALAT1 as a cancer biomarker, it can be considered a therapeutic target in cancer patients as well [43]. In prostate cancer, MALAT1 leads to tumorogenesis by inducing tumoral cell proliferation. Also, MALAT1 upregulation induces migration. MALAT1 deactivation prevents cell mobility and metastasis. Highly expressed MALAT1 is associated with poor prognosis [44]. Therefore, MALAT1 can be considered a PC regulator and a prognostic and therapeutic marker. The present study aims to evaluate the elimination of changes of MALAT1 in DU-145 cells and its effects on proliferation, migration, and apoptosis.

## Materials and methods

### Bioinformatics analysis

#### Datasets

lncRNA RNA-seq data (HTSeq-Counts) from the Middle East and Iran (https://htseq.readthedocs.io/en/master/), including PC and control samples, as well as related clinical data from PC patients, were obtained using the publicly available Genomic Data Commons (GDC) data website (https://portal.gdc.cancer.gov/) and the TCGA data website (https://dcc.icgc.org/releases/current/Projects/PRAD-TCGA). Five hundred fifty-one gene expression profiling specimens were gathered, with 499 PC and 52 non-carcinoma tissues. Three hundred ninety-three PC and 42 non-carcinoma samples were included in the 435 gene expression profiling samples obtained from ICGC. The current study excluded samples with inadequate clinical data or less than 3 months OS.

#### Detection of DElncRNAs between PC and non-carcinoma tissue samples

Clinicopathological data of 397 PC patients were gathered from the TCGA database (Table 1), while clinical data from 378 PC cases were retrieved from the ICGC database (Table 2). Tables 1 and 2 provide clinical variables for both controls and patients. The R4.0.1 program was used to conduct the analysis. The trimmed mean of M-values was used to normalize and differentially evaluate the expression patterns by the edgeR program from Bioconductor [7] to find possible lncRNAs in subsequent survival analysis. The differences in lncRNA expression in PC relative to nearby normal tissues were given as log2FC with *P*-values. The criteria in this study included |log2FC| > 1 and false discovery rate (FDR) q < 0.05. Next, the pheatmap function from the R package (version 1.0.12) was used to conduct unstructured hierarchical clustering based on DElncRNA expression profiles.

**Table 1:** List of primers along with target gene and product length utilized in this research

Gene name	Primer sequence	Annealing temperature	Product length	Gene type
BAX	F: 5′-AGGTCTTTTTCCGAGTGGCAGC-3’	64 °C	234 bp	Proapoptosis
	R: 5′-GCGTCCCAAAGTAGGAGAGGAG-3’			
FAS	F: 5′-CAATTCTGCCATAAGCCCTGTC-3’	64 °C	163 bp	Proapoptosis
	R: 5′-GTCCTTCATCACACAATCTACATCTTC-3’			
P53	F: 5′-TGCGTGTGGAGTATTTGGATGAC-3’	64 °C	170 bp	Proapoptosis
	R: 5′-CAGTGTGATGATGGTGAGGATGG-3’			
SURVIVIN	F: 5′-AGAACTGGCCCTTCTTGGAGG-3’	64 °C	170 bp	Antiapoptosis
	R: 5′-CTTTTTATGTTCCTCTATGGGGTC-3’			
BCL2	F: 5′-GACGACTTCTCCCGCCGCTAC-3’	64 °C	245 bp	Antiapoptosis
	R: 5′-CGGTTCAGGTACTCAGTCATCCAC-3’			
GAPDH	F: 5′- GAAGGTGAAGGTCGGAGTC-3’	64 °C	226 bp	Endogenous control
	R: 5′- GAAGATGGTGATGGGATTC-3’

**Table 2:** Univariate Cox regression result. According to the results of the table, MALAT1 and HOXB-AS3 had the highest expression levels in PC patients

Gene	HR	z	***P***value
**MALAT1**	1.9468361	2.642442	0.0001148
**HOXB-AS3**	3.1336003	−2.942113	0.0040574
**HNF1A-AS1**	1.3543432	2.7237227	0.0064551
**PRRT3-AS1**	0.6138334	−2.665228	0.0076936
**SLC12A5-AS1**	1.9890718	2.6631698	0.0077408
**LINC00908**	0.5863954	−2.642943	0.0082189
**FGF14-AS2**	0.5512475	−2.61246	0.0089893
**GAS1RR**	0.5758461	−2.552864	0.0106841
**LINC01679**	0.5425557	−2.53287	0.0113133
**LINC02562**	0.7342301	−2.339389	0.0193153
**LINC00342**	2.2694645	2.2884211	0.022113
**LYPLAL1-DT**	0.4522109	−2.247201	0.0246272
**PTOV1-AS2**	2.6219354	2.2380058	0.0252207
**LCMT1-AS2**	1.4869506	2.2249907	0.0260819
**UBE2R2-AS1**	1.7573767	2.2163771	0.0266657
**SNHG20**	3.0420949	2.1916125	0.0284075
**LINC00637**	2.1254849	2.1859859	0.0288166
**FLJ45513**	1.8724394	2.1835555	0.0289949
**UCKL1-AS1**	1.7855687	2.1177874	0.0341931
**LINC01018**	0.6580459	−2.107108	0.0351083
**C9orf170**	0.5338195	−2.10117	0.035626
**LINC00514**	1.4409914	2.0985571	0.035856
**C3orf35**	2.1205686	2.0971187	0.0359831
**LINC01475**	1.5466481	2.0844886	0.0371157
**LINC00668**	0.8037835	−2.070053	0.0384474
**VPS9D1-AS1**	2.2948431	2.063153	0.0390981
**LINC00398**	0.5357539	−2.043213	0.0410314
**LINC00900**	0.496077	−2.041876	0.0411638
**LINC00624**	1.9622916	2.0187215	0.0435162
**LINC01694**	1.5124827	2.013401	0.0440725
**JARID2-AS1**	0.5494942	−2.009755	0.0444571
**SNHG12**	2.0951052	2.0007556	0.0454187
**LINC01088**	0.7066184	−1.99579	0.0459568
**LINC01558**	0.6257669	−1.984459	0.0472046
**TRHDE-AS1**	0.6166486	−1.972484	0.0485544
**PCAT7**	2.2286568	1.9617039	0.049797

#### Gene ontology (GO) and Kyoto encyclopedia of genes and genomes (KEGG) pathway analyses

Then, expression profiles and sample survival of lncRNA were investigated. For differentially expressed lncRNAs, univariate and multivariate COX regression analyses were used to construct a lncRNA gene model. Next, ICGC database data were merged to assess the accuracy of the prediction model. Our model underwent survival analysis, ROC curve analysis, patient risk heat plot, risk curve, and survival status plot. Co-expression was used to suggest lncRNA target genes, then examined using functional clustering analysis. Finally, the independent prognostic analysis was performed, an association study between our lncRNA nomogram and other standard clinical features, and stratified and combined analyses of the Gleason Score. Pearson correlation coefficients between lncRNA expression and PCGs were evaluated using the z-test and two-sided Pearson correlation coefficients to find relevant biological processes and linked pathways involving predictive lncRNA. PCGs with |Pearson correlation coefficient| > 0.40 and *P* > 0.01, on the other hand, were shown to have a positive or negative connection with lncRNAs. The Database for Annotation, Visualization, and Integrated Discovery was used to conduct GO and KEGG studies on the lncRNA-related PCGs with a false discovery rate (FDR) of q < 0.05.

### Samples and cell culture

The cells DU-145 relevant to PC were provided by the Pasteur Institute (Tehran, Iran). This study has received approval from the research ethics committee of the college (IR.IAU.M.REC.1399.010). DU-145 cells in RPMI 1640 medium inclusive 1% penicillin / streptomycin antibiotics and 10% FBS in 5%CO2 and 37 °C were cultured. The culture medium was examined every 2 days to reach a cell concentration of 70-80%, during which time the cells were washed with PBS, and a new medium was added to them. The percentage of survival of cells in initial cells used for PX459, pX459-MALAT1-sgRNA1, 2, and blank control groups was 72, 78, and 80% respectively.

### Design of MALAT1 CRISPR/Cas9 vector and cell transfection

MALAT1-special sgRNAs were designed utilizing chop-chop software. The stem cell technology research center (Tehran, Iran) prepared Cas9 (pSPCs9 [BB]-2A-Puro [px459]) vectors and sgRNA oligonucleotides for our research. In MALAT1 exon1, sequences of sgRNA were designed. These sequences include sgRNA-1-F: 5′-ACTTCTCAACCGTCCCTGCA-3′ and sgRNA-2-R: 5′-GGCAGTACAAAATCTTTGGA − 3′. To disrupt the MALAT1 function and delete the desired sequence, two gRNA were designed that cut simultaneously DNA sequence in two sites. pX459-MALAT1-sgRNA1 and pX459-MALAT1-sgRNA2 vectors were used for MALAT1 knockout. Following the protocol, these vectors were transfected in PC cells via lipofectamine 3000 (Invitrogen). The positive control group was not genetically manipulated. PX459 plasmid of unmodified was utilized as the negative control. The cell screening was done with 2 μg/mL puromycin in the RPMI medium. Then cells were selected after three generations.

### Reverse transcription polymerase chain reaction (*RT-*PCR)

In order to extract DNA from DU-145 cells, a DNPTM kit (Sinaclon, Tehran, Iran) was used according to the kit instructions. Using PCR, the expression of the MALAT1 gene was assessed in transfected DU-145 cells by pX459-MALAT1-sgRNA1, pX459-MALAT1-sgRNA2, and the sgRNAs correct function. If allele deletion has been done properly, the PCR product should be shorter than the wild-type allele. Therefore, 63 °C was considered for MALAT1 as a suitable temperature. The proprietary primers of MALAT1: MALAT1-F: (5′-AGCTCTGTGGTGTGGGATTGAG-3′) and MALAT1-R: (5′-CGTTAACTAGGCTTTAAATGACGCAATTC-3′). GAPDH-F: (5′-GAAGGTGAAGGTCGGAGTC-3′) and GAPDH-R: (5′-GAAGATGGTGATGGGATTC-3′). The cycling conditions were: 95 °C for 5 min, 30 cycles of 94 °C for 40 s, 63 °C for 40 s, 72 °C for 40 s, and 72 °C for 5 min for duplication of unfinished fragments. After the reaction, PCR products underwent electrophoresis in agarose gel and were observed via UV light. Then gene fragments related to MALAT1 were cut from the gel. PCR products were purified by Favor Prep TM GE /PCR Purification kit (FAVORGEN, Taiwan) and were sent to Takapou-zist company for sequencing.

### CCK-8 analysis

DU-145 control cells and cells expressing lncRNA MALAT1 knockout were seeded at a concentration of 10^4^ cells per well in a 96-well microplate and cultivated for 7 days. Cell proliferation and viability were assessed daily. Cell viability was evaluated using a 450 nm optical plate reader after 10 mL of CCK-8 reagent (Dojindo Laboratories) was introduced to each well.

### Colony formation analysis

In 6-well plates, 800 DU-145 control cells and lncRNA MALAT1 knockout DU-145 cells were seeded and cultured for 2 weeks in full media. The cells were fixed with paraformaldehyde and stained with 0.1% crystal violet after removing the media. Finally, photographs of the labeled cells were obtained, and the number of colonies in each well was estimated.

### MTT assay

MTT assay was applied to measure cell proliferation and viability. This assay was performed according to Cell Proliferation Kit I (MTT) (Merck, Germany) as follows: Control and transfected cells inside plate 96-well were implanted. The cells were cultured at 24 h, 48 h, and 72 h. In each of the wells was added MTT reagent (Merck, Germany) and for 4 h was incubated. Also, DMSO was used to reaction stop then the cells at 2 h were incubated at room temperature and in the dark. Finally, Cell proliferation was investigated using Elisa Reader (BioTek, U.S.A).

### Cell cycle analysis

MALAT1-stably expressing DU-145 cells (Normal cell) and lncRNA MALAT1 knockout DU-145 cells were fixed in 100% ethanol for 24 h. The cells were washed twice in PBS and dyed for 15 min with BD Bioscience Pharmingen’s PI/RNase labeling buffer. FACS flow cytometry was used to examine the DNA content of the cell population. Flowjo V10 software evaluated cell cycle data (Tree Star, Ashland, OR).

### Apoptosis detection

#### Apoptosis flow cytometry analysis

Flow cytometry analysis investigated cell transfection effects on cell death and apoptosis by Annexin V Apoptosis Detection Kit (MabTag GmbH, Germany) as follows: Two days after the transfection, DU-145 cells were cultured in a six-well plate. Then digestion was done using trypsin. Cell lines were washed using PBS and were centrifuged for 5 min at 1000 rpm. The binding buffer was used based on the protocol to regulate cell concentration. From staining solutions of Annexin V-fluorescein isothiocyanate (FITC) and propidium iodide (PI), 5 μl in 200 μl of samples were added. Then samples were mixed and incubated in the dark for 15 min at room temperature. Finally, the Cell cycle analysis was done through flow cytometry (PARTEC, Germany).

#### Apoptosis genes analysis using quantitative RT-PCR

The extraction process of total RNA was done from cell samples using RNX-PlusTM (Cinnagen, Tehran, Iran). The concentration of RNA was evaluated by a Nanodrop spectrophotometer (Varian, Australia) at 260/280 nm wavelength. Then, cDNA was synthesized by the cDNA Synthesis kit (Yekta Tajhiz Azma, Tehran, Iran). Using qRT-PCR, the expression level of anti-apoptotic and pro-apoptotic genes was determined in DU-145 transfected and non-transfected cells by SYBR Premix Ex Taq™ kit (Takara Bio, Japan). The characteristics of the genes used are demonstrated in Table 1. GAPDH gene was selected as endogenous control. The cycling conditions inclusive: initial denaturation at 95 °C for 5 min, 30 cycles for denaturation at 94 °C for 40 s, annealing at 64 °C for 40 s, and extension at 72 °C for 40 s, followed by 72 °C for 5 min for duplication of unfinished fragments. Analysis of gene expression was performed using (2^-ΔΔCt^) method.

### Cell migration assay

The cell scratch assay was utilized to analyze cell migration. In a plate of six-well, both control cells and transfected were cultured to reach a concentration of 80%. After cells spread, they were washed using PBS. Cell scratch was created with a P10 pipette, and migration was investigated every 6 h. Ulcer size was observed under an inverse light microscope.

### Statistical analysis

The data was drawn by Tukeys test and GraphPad Prism software (GraphPad, San Diego, California) version 7. Results were reported as means ± SD. Statistical analysis was done using SPSS software version 20 (SPSS, Inc., Chicago, IL, USA). *P*-value < 0.05 was considered significant differences amongst experiment groups using t-test and one-way ANOVA.

## Results

### DElncRNAs were found in PC and non-carcinoma tissue samples

**Fig. 1:**
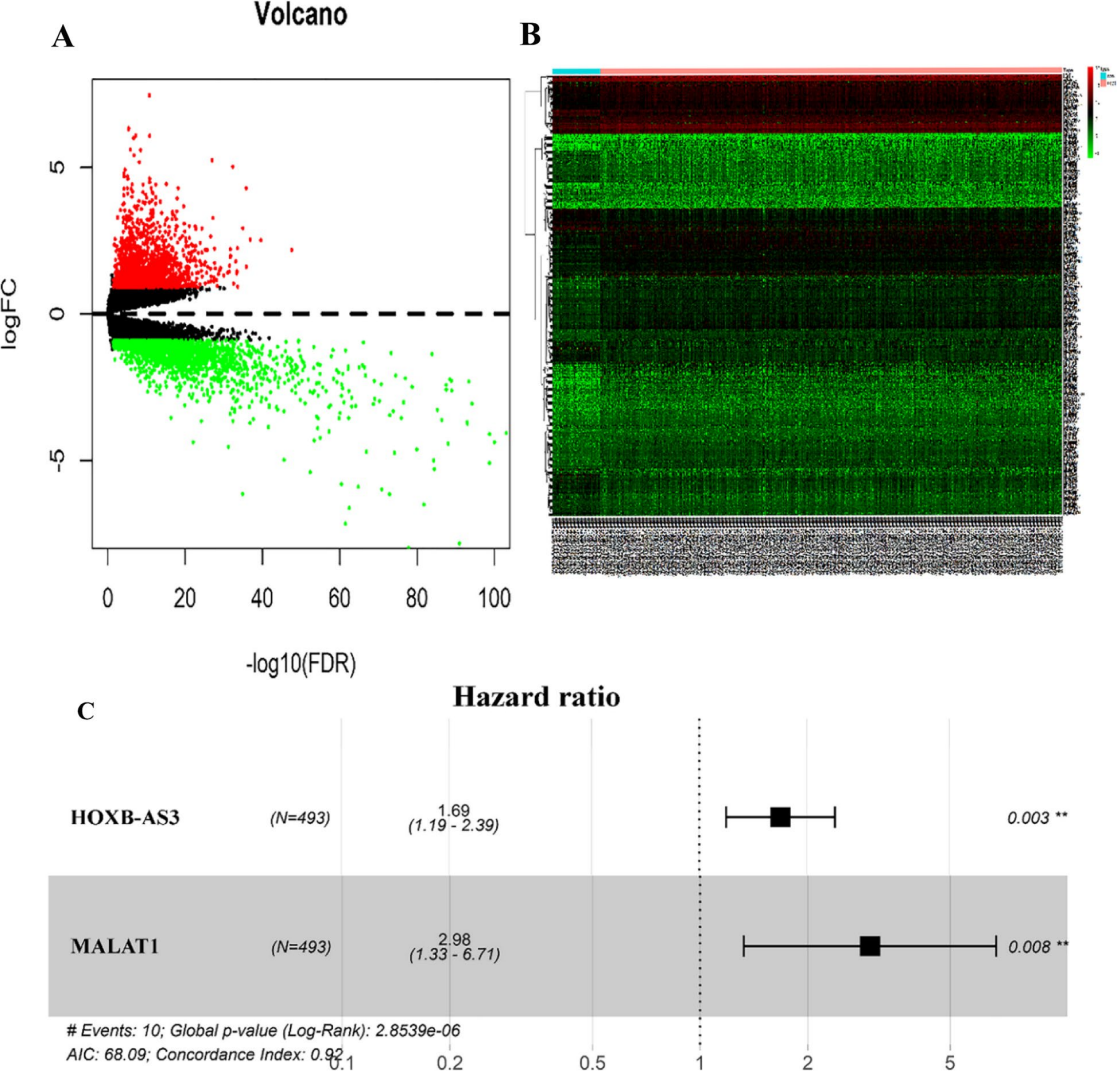
**A** Prostate tumors and non-carcinoma tissue samples have different DElncRNAs. Differentially elevated lncRNAs are shown by red dots, down-regulated lncRNAs are represented by green dots, and black dots represent no genes. **B** The differentially expressed lncRNAs in prostate cancer and normal tissues were studied using unsupervised hierarchical clustering. **C** Multivariate Cox regression analysis generated a forest map of 2 lncRNAs

Predicated on thresholds of |log2FC| > 1.0 and false discovery rate (FDR) q < 0.05, a maximum of 451 lncRNAs were discovered to reflect different expressions between PC and non-carcinoma tissue samples, with 307 being upregulated and 144 being down-regulated, and they were used in later stepwise survival analysis (S1-Table). Volcano graphs naturally portrayed expression characteristics (Fig. 1A). The unstructured hierarchical cluster analysis was carried out based on the DElncRNAs patterns, which revealed the capability of distinguishing between PC and non-carcinoma specimens (Fig. 1B). The lncRNA expression patterns were assessed using univariate Cox appropriate statistical analysis to detect prognosis-related lncRNAs linked with patients’ OS in PC patients. At a 0.05 threshold, 36 lncRNAs related to OS were carefully selected (Table 2), which were then subjected to stepwise multivariate Cox regression analysis, with 2 lncRNAs (MALAT1, HOXB-AS3) (as shown in Fig. 1C, Table 3) eventually being screened out of the 451 lncRNAs identified previously to generate a predictive model.

**Table 3:** A summary of the 2 prognostic lncRNAs linked to PC. MALAT1 and HOXB-AS3 eventually being screened out of the 451 lncRNAs identified previously to generate a predictive model

Gene	Coef^**a**^	Exp [coef]^**b**^	Se [coef]^**c**^	z	Pr [> |z|]
**HOXB-AS3**	−0.524052278	1.688857522	0.178121024	−2.942113552	0.003259804
**MALAT1**	1.092940489	2.983032765	0.413609893	2.642442815	0.008231036

^a^*Coef* coefficient

^b^*Exp [coef]* hazard ratio

^c^*Se [coef]* the range of values at hazard ratio

**Fig. 2:**
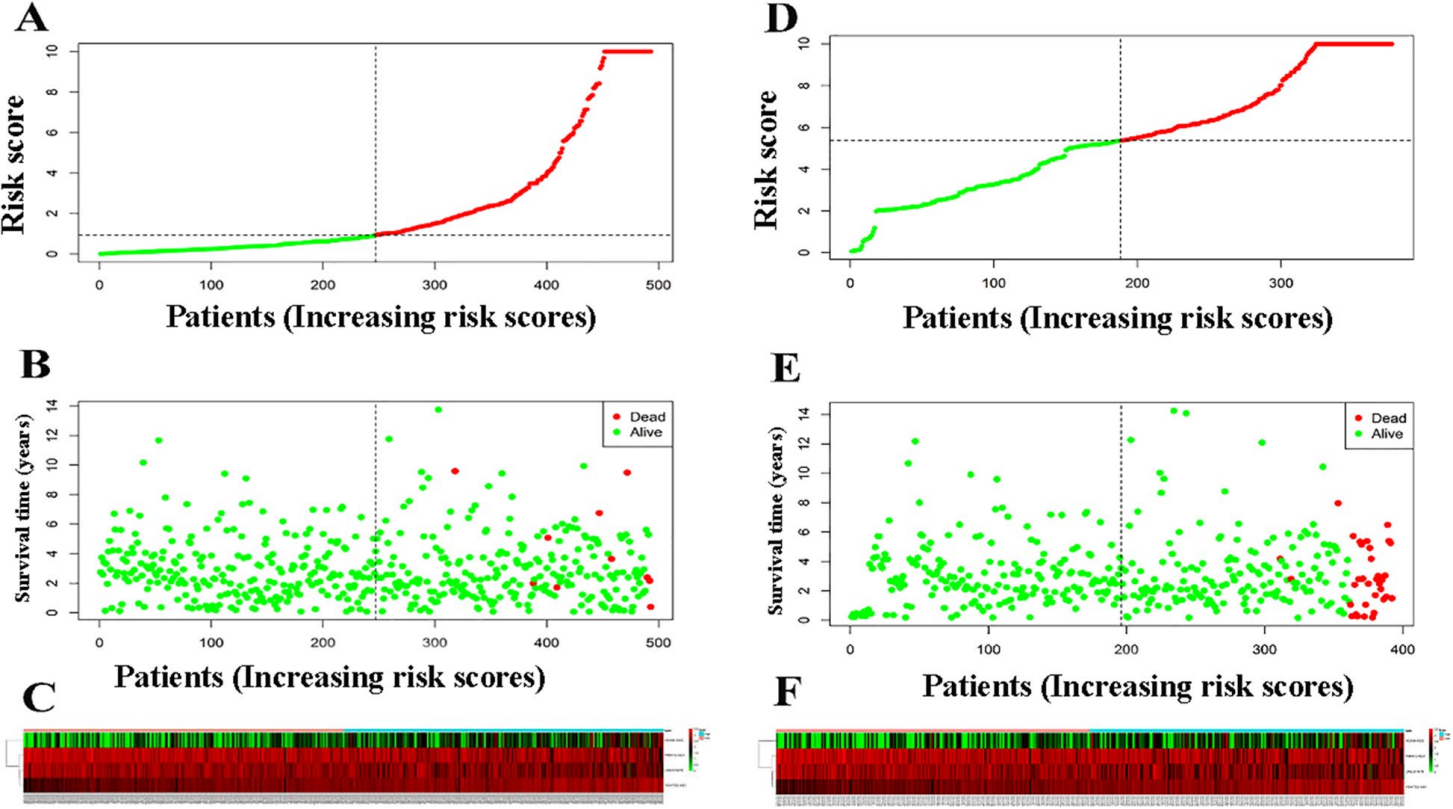
The ability of two lncRNAs based on the signature to predict prognosis for PC patients from the TCGA database. **A** Patients’ risk scores are distributed. **B** The average length of time for a PC patient to survive. **C** A heatmap of the expression levels of the 2 lncRNAs used in the prognostic model. The ideal threshold for dividing patients into high- and low-risk groups are represented by the vertical black dotted line. The model’s prognostic predictive power in patients with PC. **D** Patient risk score distribution. **E** PC patients’ survival time. **F** Heatmaps of the prognostic model of two lncRNAs. The appropriate threshold for categorizing instances into high- and low-risk categories is represented by the vertical dotted black line

A prognostic nomogram was produced by combining the expression patterns of lncRNAs with the associated regression coefficients. The survival risk score was calculated using the following formula: survival risk score = (0.524052278 HOXB-AS3 expression) + (1.092940489 MALAT1 expression). MALAT1 had positive coefficients on multivariate Cox regression analysis, associated with high risk because the upregulated level indicated a reduced patient OS (MALAT1, Coef > 0). In contrast, the remaining HOXB-AS3 lncRNAs (HOXB-AS3, Coef < 0) had negative coefficients, indicating the protective role due to up-regulation of lncRNAs to show extended OS relative to patients having decreased expression (Fig. 1C). According to the median risk score (0.943) calculated from the two lncRNA expression levels (also known as the survival risk score, SRS) in the TCGA database, the 493 samples were categorized as high- (*n* = 246) or low-risk (*n* = 247) (Fig. 2A, B, C, S1-Table and S2-Table).

**Fig. 3:**
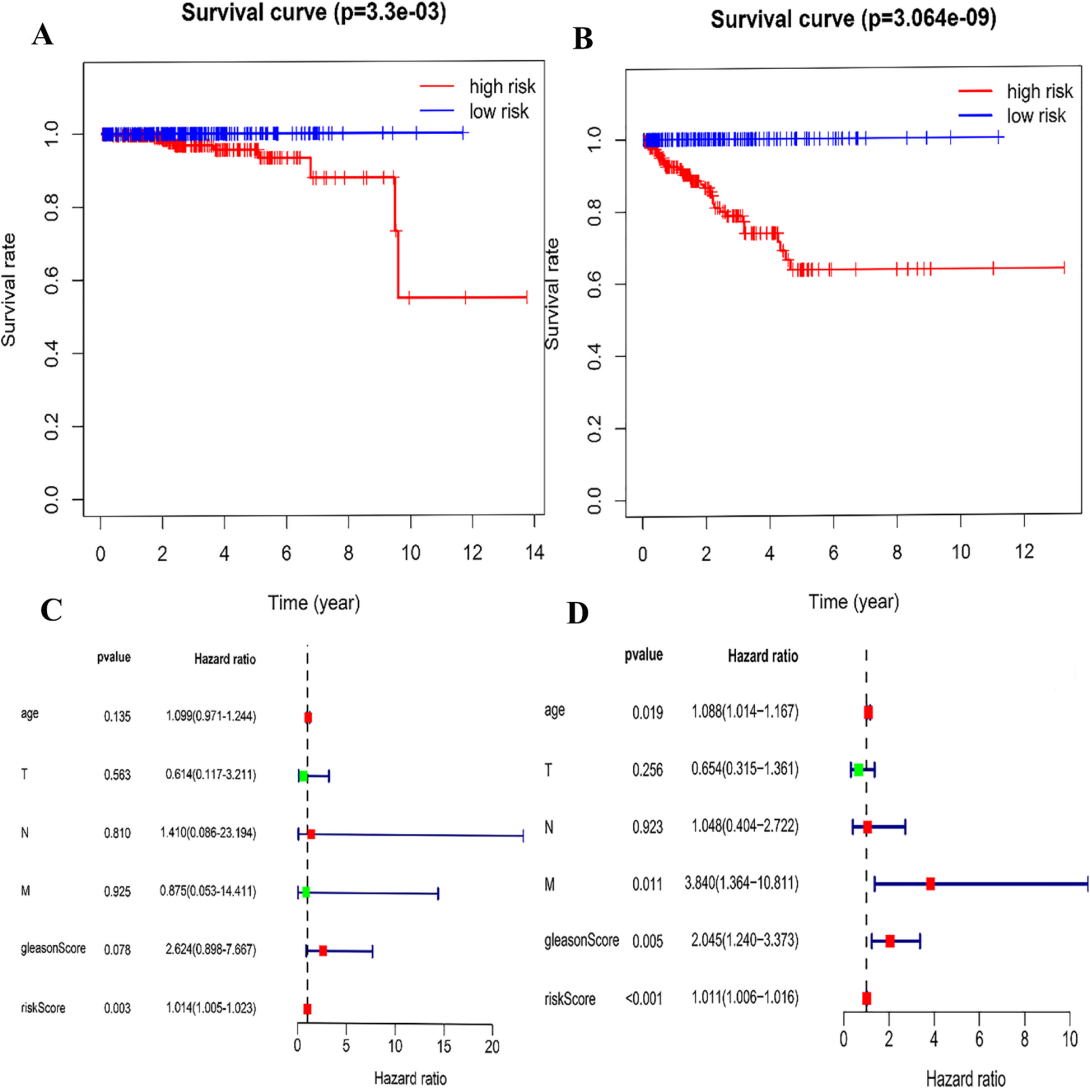
**A** Based on TCGA data, the two-lncRNA nomogram’s prediction capacity was assessed using Kaplan-Meier analysis, log-rank P, and C-index. The Kaplan-Meier curves were used to depict and compare OS time between low- and high-risk groups. **B** The predictive ability of two-lncRNA nomograms based on ICGC data was assessed using Kaplan-Meier analysis, Log-rank P, and C-index. **C** The findings of the TCGA multivariate analysis showed that the 2-lncRNA signature might be used as a powerful predictor of PC OS rate when compared to other clinicopathological variables. **D** The findings of the ICGC multivariate analysis revealed that 2-lncRNA features might be utilized to predict PC OS rate in the absence of other clinicopathological variables

**Fig. 4:**
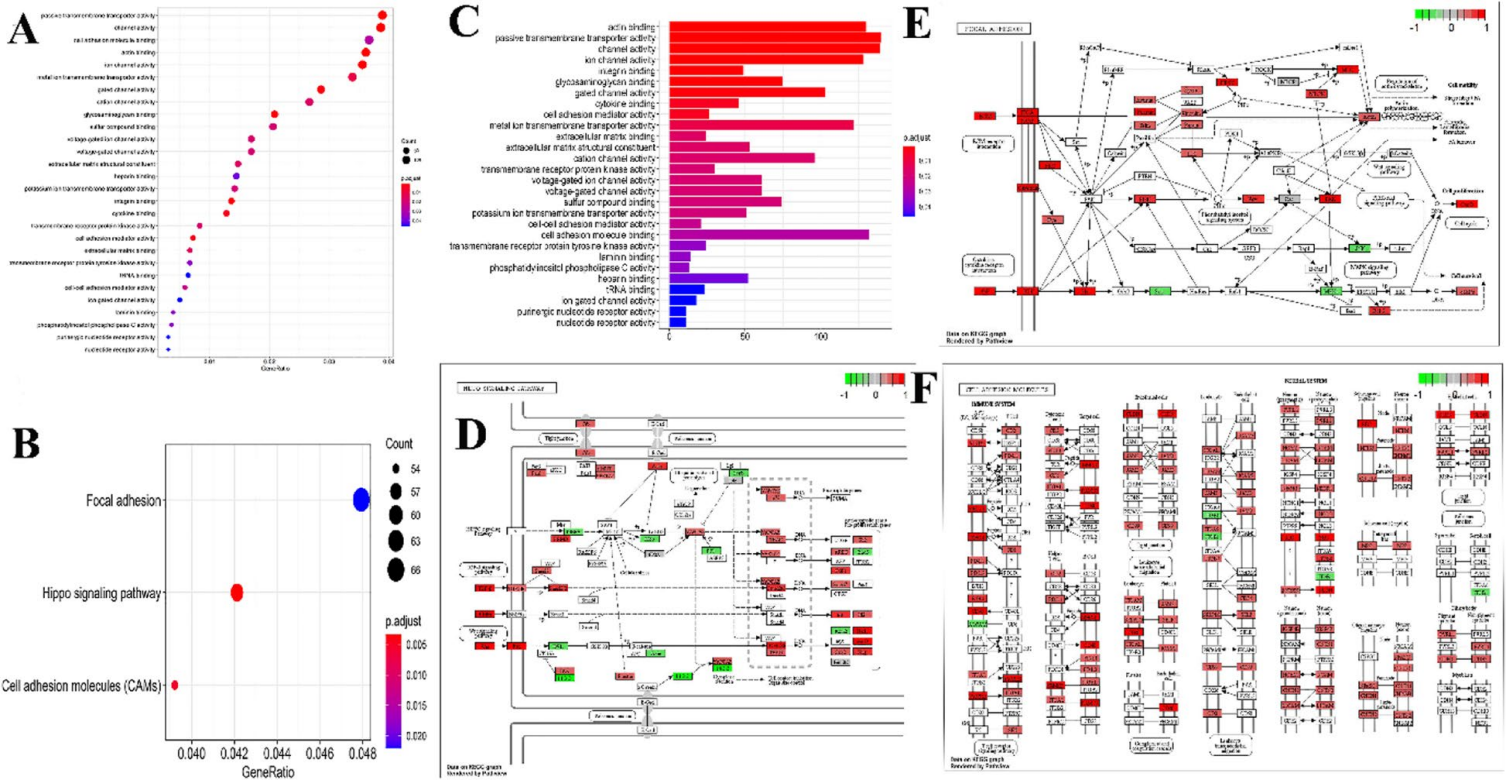
Pathway enrichment study of the biological processes and contribution with the MALAT1 lncRNAs employed in the model. **A** GO enrichment results in biological processes. **B** An investigation of the KEGG signaling pathways. **C** Histogram of GO keywords for MALAT1-lncRNA-related PCGs based on enrichment analysis. **D** MALAT1-lncRNAs-related PCGs KEGG enrichment pathway maps, Hippo signaling pathway Focal adhesion molecules (**E**) and cell adhesion molecules (**F**) are two types of adhesion molecules [CAMs]

The median SRS was 1.561 according to the expression profiles of these two lncRNAs in the ICGC database, and 392 patients were separated into two groups: high (*n* = 196) and low (n = 196) (Fig. 2D, E, F, and S-3 Table). The log-rank test was used to detect the survival difference. The K-M approach was applied for survival analysis. Univariate and multivariate analyses were used to evaluate the independent prognostic value of the model of our developed 2 lncRNAs-based signature of the survival risk for PC patients after considering other possible conventional prognostic markers. The multivariate results showed that our 2 lncRNA-based signature could be used to predict PC OS rate from other clinical characteristics independently (TCGA hazard ratio (HR) = 1.014, 95% Ci 1.005–1.023, *P* = 0.003; ICGC HR = 1.011, 95% Ci 1.006–1.016, *P* > 0.001), as shown in Fig. 3, when compared to conventional clinicopathological factors such as age, Gleason score, and TNM classification. This study selected co-expression between lncRNAs MALAT1 and 4080 PCGs (|Pearson correlation coefficient| > 0.4, *P*-value < 0.05, and q-value 0.05 < 0.05) after establishing the relationships between those 2 lncRNAs and the PCGs. Then, for the lncRNA-related PCGs, GO, and KEGG analyses were performed to show the potential roles of lncRNAs MALAT1 in cancer development. The 4080 PCGs were enriched in 28 GO keywords and were found in the extracellular matrix (ECM) and cell membrane, with most of their activities related to adhesion, activation, and transport of chemicals across the ECM and cell membrane (Fig. 4A, B and C). Three KEGG pathways were found to be overrepresented, all of which are associated with cell contact and binding, as well as cell proliferation (hsa04390: Hippo signaling pathway, hsa04514: Cell adhesion molecules (CAMs), and hsa04510: Focal adhesion) (Fig. 4D and E, F). As a consequence of the findings of bioinformatics investigations, lncRNA MALAT1 has been identified as a possible candidate in the development and progression of prostate cancer. As a result, it is crucial to investigate the lncRNA Knockout and its consequences.

### LncRNA MALAT1 increases proliferation of DU-145 human prostate cancer cell lines

**Fig. 5:**
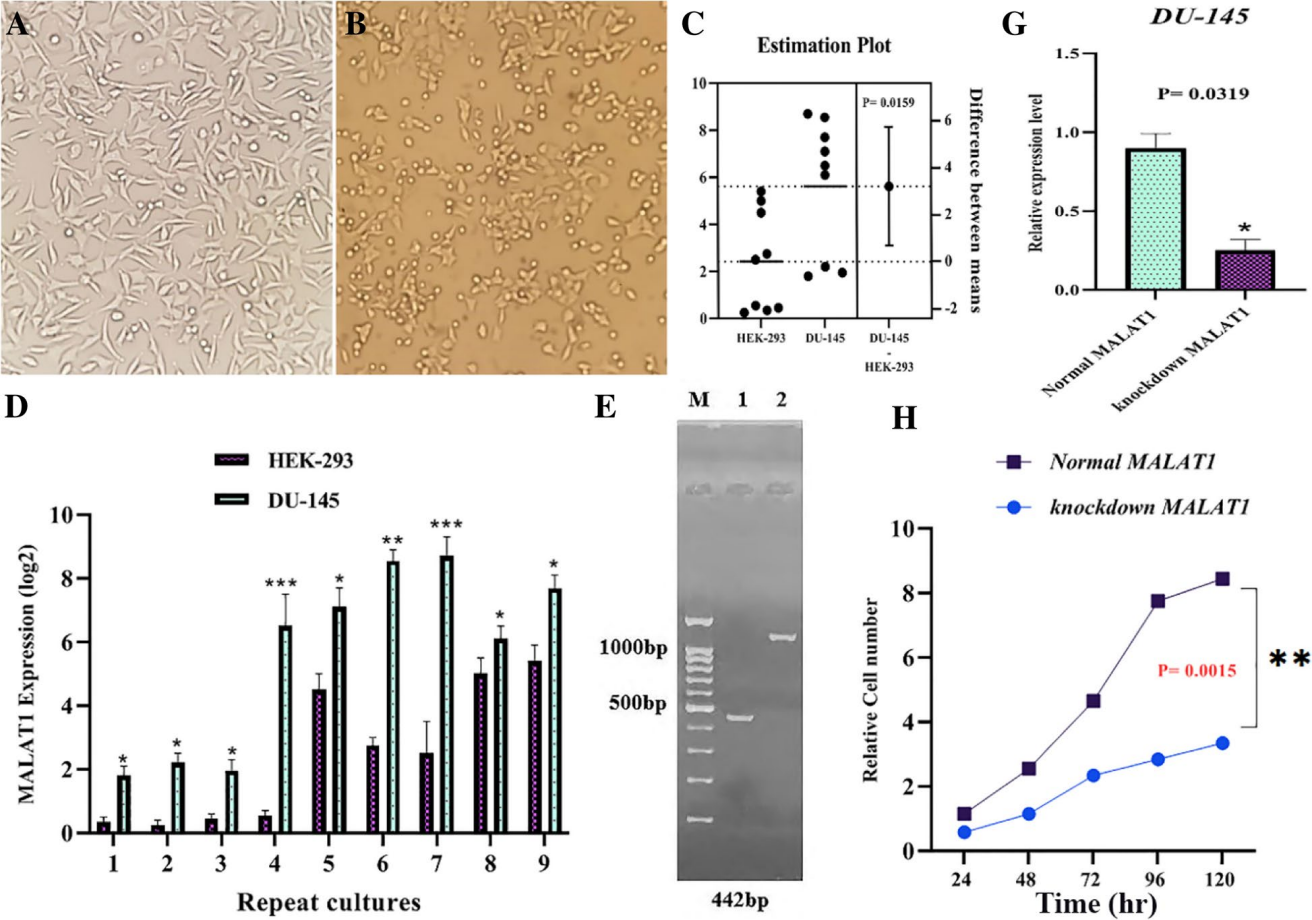
**A**, **B** MALAT1 expression in DU-145 control cells and the transfected cells. **C**, **D** lncRNA MALAT1 is highly expressed in human PC cell carcinoma. **E** Products of RT-PCR in DU-145 transfected cells and non-transfected cells for expression appraise of MALAT1 gene on the agarose gel. M: molecular marker of DNA, 1: amplified fragments of MALAT1 [442 bp] in DU-145 transfected cells, 2: MALAT1 expression [1133 bp] in DU-145 non-transfected cells [control cells]. **G** LncRNA MALAT1 increases the proliferation of PC cell lines. Norma MALAT1 and knockdown-MALAT1 were transfected into DU-145 cells. The knockdown efficiency of MALAT1 was detected by RT-PCR. **H** DU-145 cells expressing MALAT1 cells and Normal MALAT1 were cultured in 96-well plates, and cell viability was measured using CCK-8 after 1, 3, and 5 days of culture. *P < 0.05, **P < 0.01, ***P < 0.001

According to studies in this field, MALAT1 acts as an oncogene in prostate cancer cell lines, so its expression level is considerably higher in these cells (Fig. 5). The impact of MALAT1-lncRNA was examined on PC cell line proliferation. RT-PCR was used to investigate the expression of MALAT1-lncRNA in DU-145 human cell lines and human embryonic kidney 293 cell line (Positive control). MALAT1-lncRNA was the most prevalent in the DU-145 cell lines (Fig. 5C, D). Thus, the DU-145 PC cell line was used as a study model for knocking out MALAT1-lncRNA. After transfection of DU-145 cells via vectors (Px459-MALAT1-sgRNA1, Px459-MALAT1-sgRNA2), sgRNAs function was investigated in transfected cells compared to control by PCR. The results demonstrated the highly efficient sgRNAs performance. The separation of products on agarose gel demonstrated fragments with the length of 442 bp for the MALAT1 gene in DU-145 transfected cells. These fragments are not found in blank control. These results acknowledge transfection correctness and vector expression in DU-145 transfected cells (Fig. 5E). Finally, the products were extracted for sequencing (Fig. 5F). lncRNA MALAT1 knockout was confirmed at the RNA level in DU-145 cells (Fig. 5G). lncRNA MALAT1 knockout inhibited DU-145 cell growth considerably (Fig. 5H). The lncRNA MALAT1 knockout inhibited the proliferation of the DU-145 cells.

### MALAT1 knockout reduces the proliferation and viability of tumor cells

**Fig. 6:**
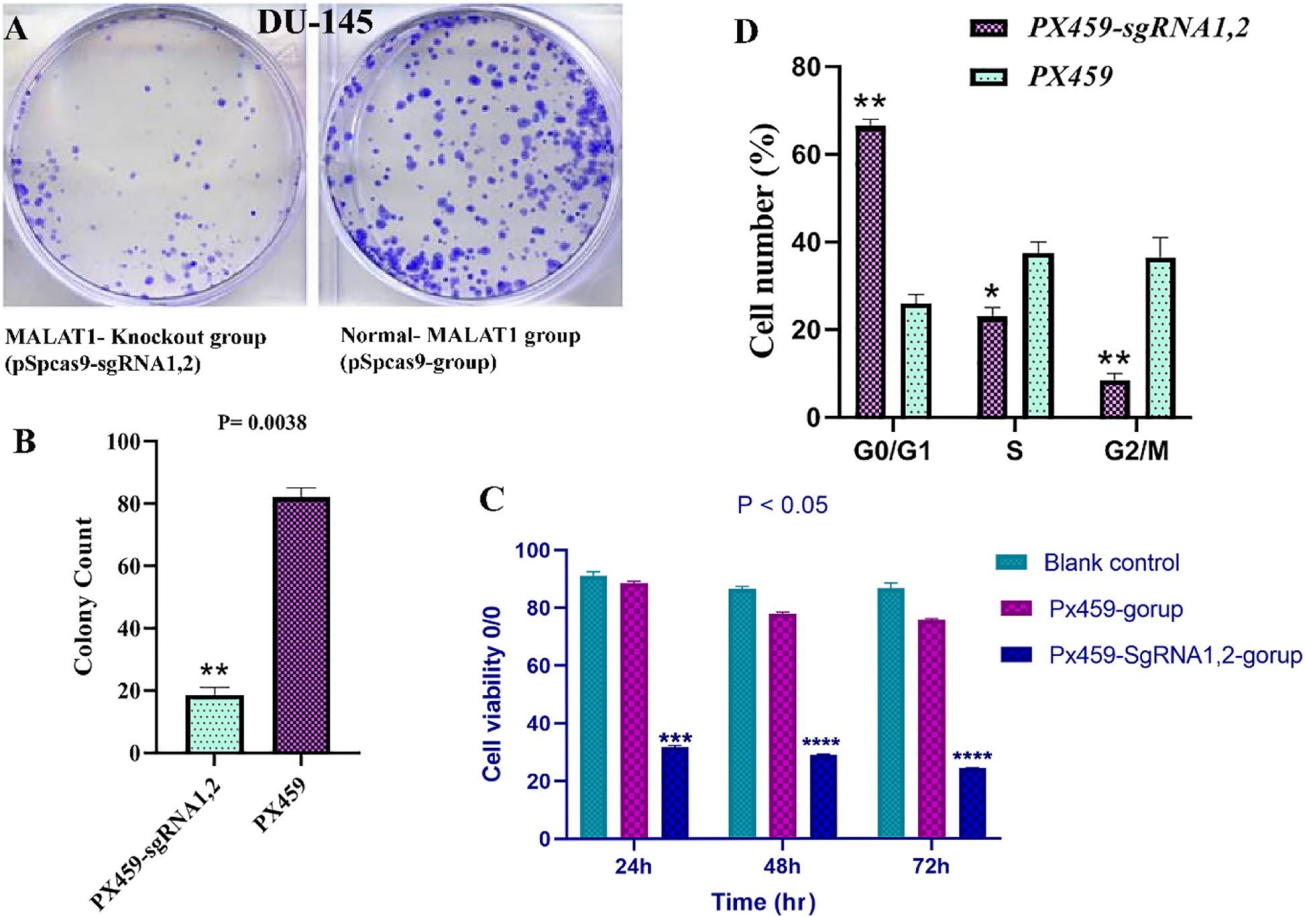
**A** DU-145 cells expressing pX459-MALAT1-sgRNA1, 2 cells, and control PX459 were cultured in 6-well plates and subjected to colony formation assays. **B** The count of the cell clones in the pX459-MALAT1-sgRNA1, 2 groups, and the PX459 groups. **C** Effect of pX459-MALAT1-sgRNA1, 2 on viability and cell proliferation of prostate cancer. Data indicates a significant difference amongst PX459-sgRNA1, 2, and blank control. *P < 0.05, **P < 0.01

The CCK8 assay was used to study the influence of MALAT1 on the proliferation of PC cell lines. Both the pSPCs9-group and the blank control of DU-145 cells had a high rate of cell proliferation. As a result, in the pSPCs9-MALAT1-sgRNA1,2 groups, knocking out MALAT1 decreased the proliferation of DU-145 cells CCK-8 assay (Fig. 6A). Colony formation assays showed that the number of clones in the DU-145 cells with MALAT1knockout by pSPCs9-MALAT1-sgRNA1,2 was reduced. The number of clones in the MALAT1-Knockout group (pSPCs9-MALAT1-sgRNA1,2) was counted to show significant statistical differences from the normal-MALAT1 group (pSPCs9-group) (Fig. 6A). Results of the present study confirmed that lncRNA MALAT1 promotes the proliferation of PC cells. The rate of cell proliferation was evaluated by MTT assay. The necrosis or and apoptosis processes reduced cell viability. Due to the linear relationship between the construction signal and cell number, the cell proliferation rate was measured (Fig. 6C).

### lncRNA MALAT1 accelerates cell cycle progression in PC cells

Cell proliferation is intimately linked to cell cycle progression. Flow cytometry was used to study cell cycle regulation in DU-145 cells expressing pX459-MALAT1-sgRNA1, 2 and PX459group vectors (Fig. 6D). MALAT1 knockout was associated with an increase in G0/G1 and a decrease in the ratio of the S phase to the G2/M phase compared to the pX459 control group. It indicated that the MALAT1 knockout inhibited cell cycle progression.

### LncRNA MALAT1-knockout increase apoptosis of PC cells

**Fig. 7:**
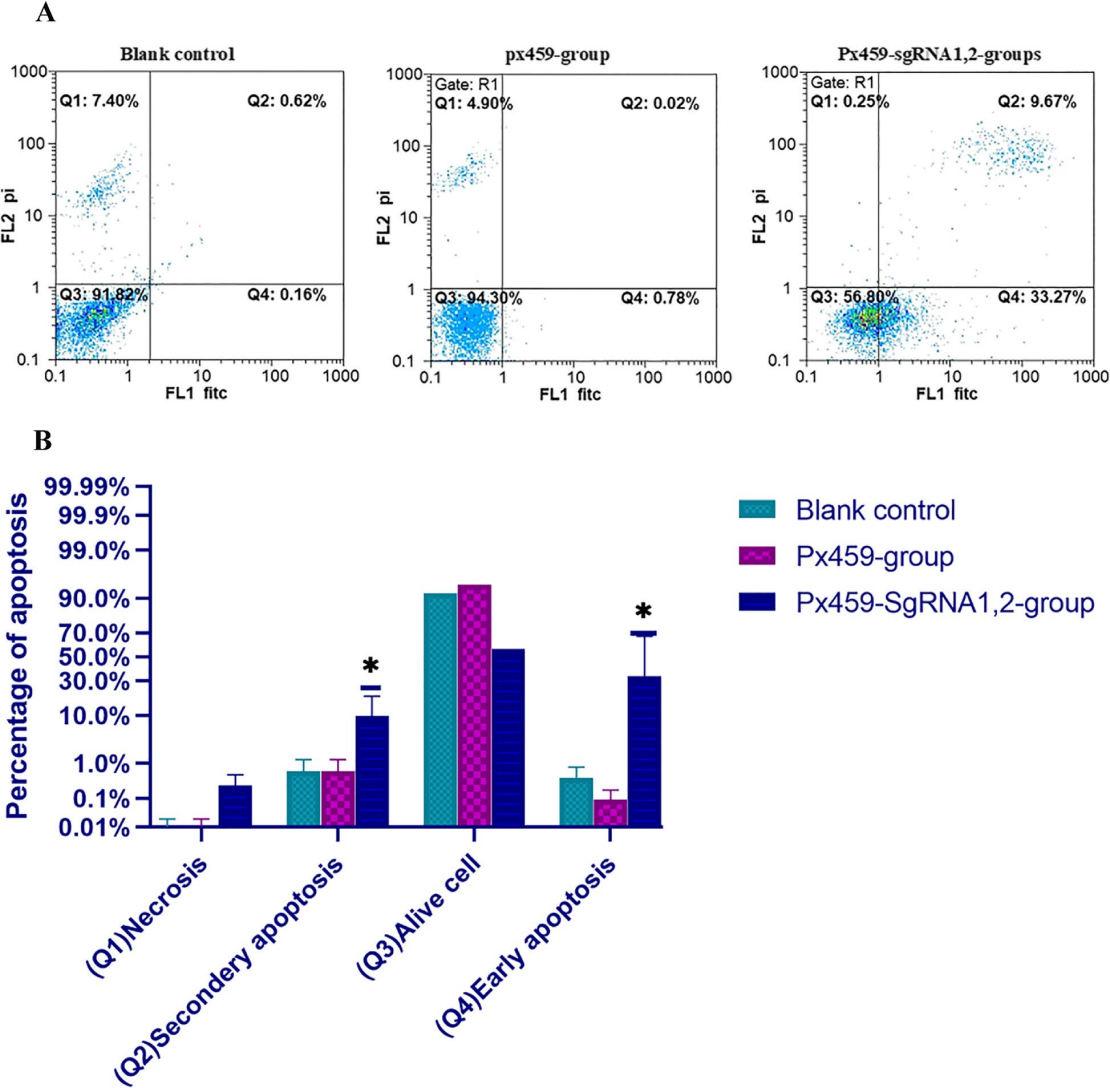
The result of apoptosis analyzes. **A** Results of flow cytometry assay. **B** Apoptotic cells percentage in each cell cycle period. *P < 0.05

The reduction of cell proliferation by PC cells expressing pX459-MALAT1-sgRNA1, 2 was accompanied by apoptosis. Flow cytometry was used to identify apoptosis in DU-145 cells that expressed pX459 and pX459-MALAT1-sgRNA1, 2 groups (Fig. 7A). The proportion of apoptotic cells in the transfected PC DU-145 cell lines was considerably higher than in the control group. About 48 h after transfection, a flow cytometry assay was used to assess the apoptosis effect on DU-145 cell lines. Data were associated with apoptosis increment. The primary and total apoptosis in the transfected group was higher than control and pX459 groups. So, MALAT1 in PC act as an oncogene via adjustment of apoptosis, cell proliferation, and migration (Fig. 7, *p* value< 0.05).

### LncRNA MALAT1-knockout increase expression of apoptosis related genes

**Fig. 8:**
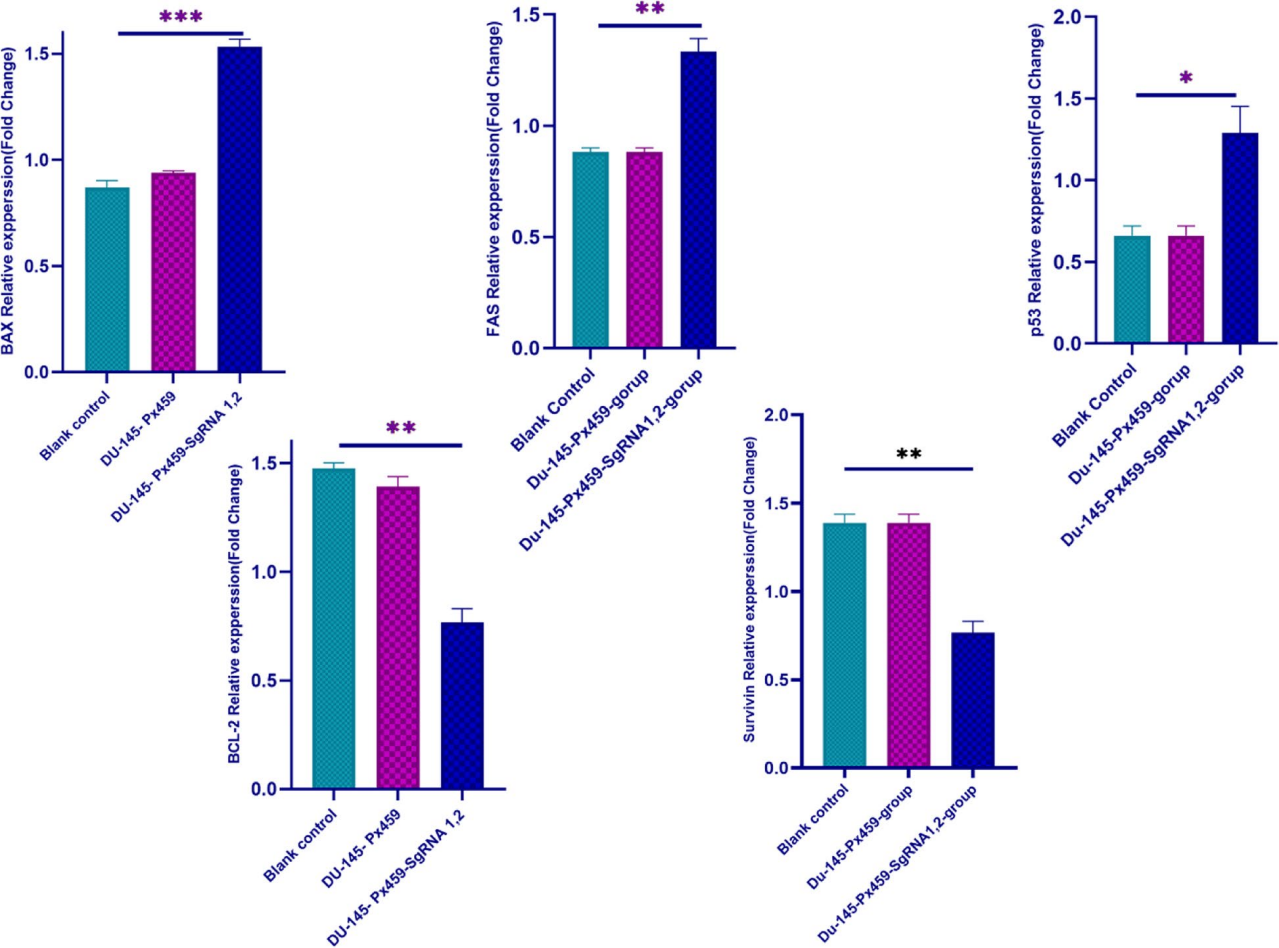
The data showed a dramatic difference in DU-145-px459-sgRNA1, 2 compared with DU-145-px459 and blank control. *P < 0.05, **P < 0.01, ***P < 0.001

Apoptosis genes expression was investigated using this method. The expression of pro-apoptotic genes *FAS*, *BAX*, and *P53* and anti-apoptotic genes BCL2 and SURVIVIN were assessed using a quantitative real-time PCR technique. As expected, pro-apoptotic genes expression was significantly higher in pSPCs9-MALAT1-sgRNA1, 2 groups compared to pSPCs9-group and blank control (Fig. 8, *p* < 0.01). Next, the expression of anti-apoptotic genes [*BCL2*, *SURVIVIN*] was assessed in DU-145 cell groups. *BCL2* and *SURVIVIN* genes expression was higher in the control cell lines (pSPCs9-group, and blank control) than in pSPCs9-MALAT1-sgRNA1,2(*p* < 0.05). Figure 8 shows that the expression of *P53*, *BAX*, and *FAS* pro-apoptotic genes increased in the DU-145 cell lines in which *MALAT1* was destroyed. Based on observations, pro-apoptosis and anti-apoptosis genes had induced and inhibited expressions, respectively (Fig. 8).

### *MALAT1* knockout suppresses cell migration

**Fig. 9:**
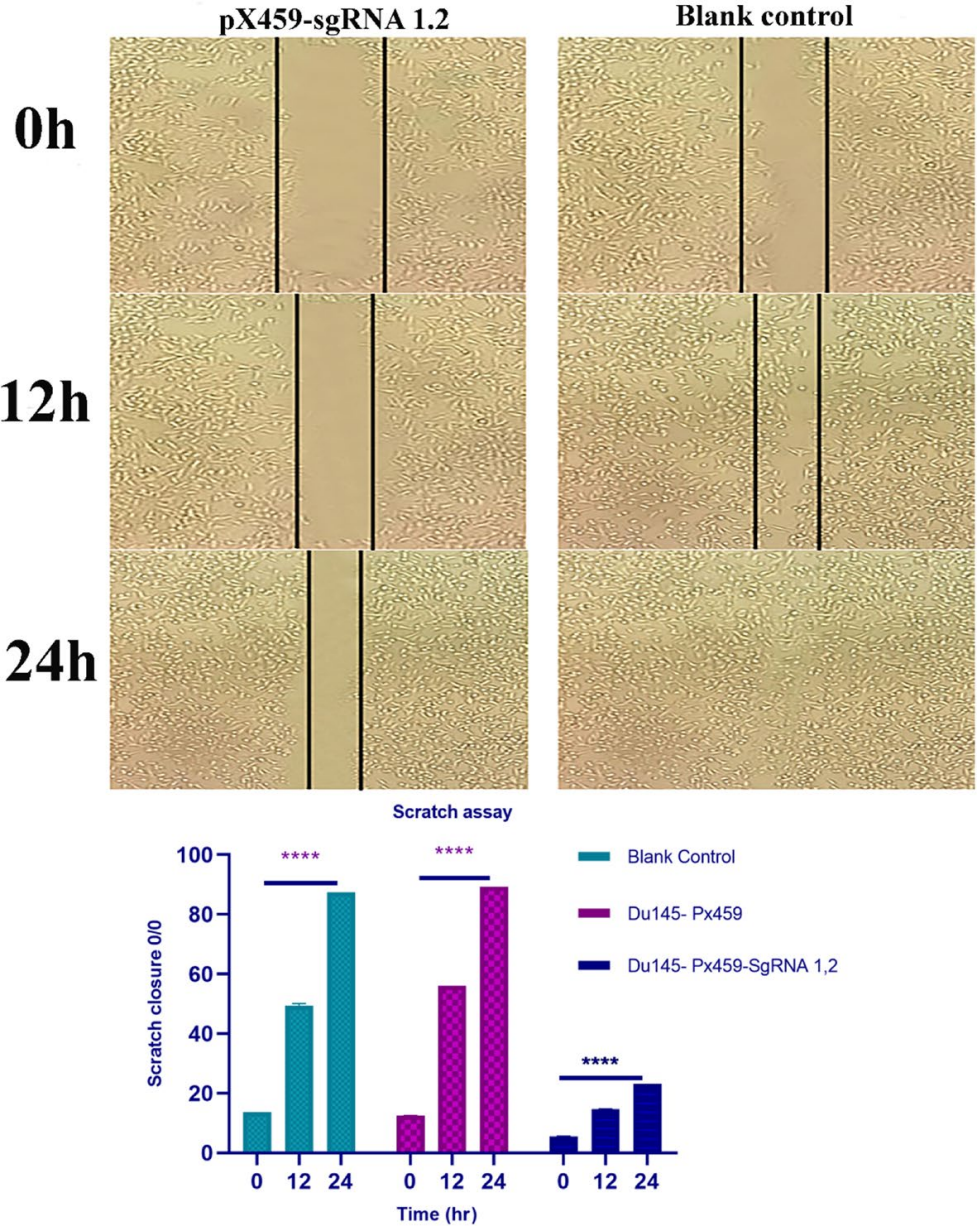
To investigate the role of MALAT1 in cell metastasis of prostate cancer, using the scratch assay was analyzed the migratory ability of PC cells. **A** Inhibition of migration in cell groups [pX459-MALAT1-sgRNA1, 2 and blank control]. Assessment of migration using a scratch assay in time intervals of 0 h, 12 h, and 24 h. **B** Data shows a quantitative percentage of cell migration inhibition. Scratch closure in the scratch assay was used as the distance of cell migration at time periods of 0-24 h. ****P < 0.0001

Assessment of MALAT1 Knockout on prostate cancer cells migration. Scratch assay was used to determine the number of migrated cells. Results showed that scratch width in transfected DU-145 cells was more noticeable than in non-transfected cells. Given that proliferation in MALAT1 knockout cells is highly reduced, the effect of decreased migration may be due to reduced proliferation (Fig. 9).

## Discussion

Cancer risk determination tools are critical for personalized clinical diagnosis and therapy. However, traditional clinicopathological variables, such as risk classification among PC patients using Gleason Score, confront significant problems [8]. As a result, it must be addressed as soon as possible to create a more sensitive and effective PC prediction model. The increasing number of lncRNA research articles has given us a new viewpoint on identifying and treating illnesses like cancer.

Using TCGA and ICGC RNA sequencing data, the maximum possible lncRNAs were found. The 2 lncRNAs-based nomogram has strong discriminating power, with AUC values of 0.997, 0.929, and 0.928, respectively, for the entire TCGA dataset at 1, 3, and 5 years. In years 1, 3, and 5, the AUC of the ICGC dataset was 0.946, 0.928, and 0.905, respectively. The as-constructed lncRNAs-based signature used in this work might be a helpful tool for PC diagnosis and treatment. Systemic or adjuvant treatment may be used in high-risk PC patients, whereas active surveillance may be used in low-risk instances. As a result, therapy evaluations will be more successful. In a univariate and multivariate COX analysis, including typical clinicopathological risk variables such as age, TNM classification, and Gleason score for PC, the 2-lncRNA nomogram (HOXB-AS3, MALAT1) was demonstrated to be linked with patient prognosis and risk stratification. As a result, it is critical to investigate the knockout of this lncRNA and its consequences. For genetic changes creation in the genome is applied gene editing. This technique simplifies gene performance study by demonstrating the biological functions of main genes. One of the gene-editing technologies is CRISPR/Cas9 that purposefully used in genome editing [45-47]. This technique could be applied to edit each gene [48, 49]. CRISPR/Cas9 is a strong technique, efficient, cheap, economical, high specificity, has a simple scheme, and can screen all genomes for functional analysis. It also identifies genes that cause particular diseases [50-52]. CRISPR/Cas9, a genetic manipulation technique, is applied for transcriptional regulation, knocking-in, and knockout. Given that gene editing is a tool for tumor therapy, CRISPR/Cas9 has become a novel technique that can be considered in tumor incidence, progress, and metastasis mechanisms. Also, this technique to the knockout of particular genes and mutation repair is used in tumor therapy [53]. Since the lncRNAs have an essential role in the beginning and advance of prostate cancer, they can be considered therapeutic targets. Many studies demonstrate that the various diseases, from hyperglycemia to cancers, have a close relationship with MALAT1 [54]. Jadaliha et al. showed that expression levels of MALAT1 do not entirely predict the regulation of invasion in breast cancer. In positive HER2 BC cells or positive ER, even with low expression levels of MALAT1, disease progression and metastasis are reported in triple-negative BC cells [55]. Lai et al. proved that MALAT1 in hepatocellular carcinoma (HCC) tissues and cells is highly expressed, and MALAT1 knockdown inhibits migration, invasion, and proliferation but induces apoptosis [56]. In cervical cancer (CC), Yang et al. found that MALAT1 is associated with tumor size and stage, lymph node metastasis, and vascular migration. Also, it is highly expressed in HPV infection [57]. Therefore, it can be an independent biomarker of cancer prognosis. MALAT1 knockdown decreases cell migration and cell invasion and is a therapy and prognosis factor in CC [57]. Xiang et al. demonstrated that increased apoptosis and decreased mobility and tumor factor expression are related to silencing MALAT1 in glioma cells [58]. Fan et al. showed which MALAT1 in cells of bladder cancer is related to increment of invasion and metastasis and promotion of EMT. Also, MALAT1 expression correlated with E-cadherin expression reversely. Therefore, MALAT1 knockdown can be a potential therapy for bladder cancer [59]. Ren et al. have shown increased expression of MALAT1 in PC cell lines. Decreased MALAT1 expression suppresses migration and invasion. Also, cell growth is arrested in G0/G1 phase. Therefore, it shows the role of MALAT1 in tumorigenesis and may be applied as a clinical treatment [44]. Wang et al. specified the urine MALAT1 as a predictive biomarker of PC that can be used instead of traditional measurements of PSA [60]. Aiello et al. demonstrated that MALAT1 suppresses receptor genes of sex steroid hormones related to PC like PSA and PS2. So, MALAT1 is considered a PC predictive biomarker [61]. In a study, Azadeh et al. stated that NEAT1 can be a diagnostic biomarker in breast cancer and stomach cancer patients by targeting XIST, hsa-miR-612, and MTRNR2L8. In accordance with the above study, our study showed that research on lncRNAs can contribute to cancer prognosis [62].

## Conclusion

Given the increased expression of MALAT1 in prostate cancer cells, MALAT1 knockout by CRISPR/Cas9 demonstrated that Px459-MALAT1-sgRNA1 and pX459-MALAT1-sgRNA2 vectors inhibited the development and proliferation of prostate cancer DU-145 cells. Since the MALAT1 can facilitate cancer advancement with the regulation of gene expression, the expression of apoptosis genes was assessed in DU-145 cells. The data confirm increased expression of pro-apoptotic genes in transfected cells compared with non-transfected cells. As a result, vectors induce necrosis and apoptosis and inhibit migration. Therefore, MALAT1 can be considered as a tumor biomarker in diagnosing and treating PC.

## Supplementary Information

**Additional file 1.**

**Additional file 2.**

**Additional file 3.**

**Additional file 4: Figure S1.** (A) Prostate tumors and non-carcinoma tissue samples have different DElncRNAs. Differentially elevated lncRNAs are shown by red dots, down-regulated lncRNAs are represented by green dots, and black dots represent no genes. (B) The differentially expressed lncRNAs in prostate cancer and normal tissues were studied using unsupervised hierarchical clustering. (C) Multivariate Cox regression analysis generated a forest map of 2 lncRNAs. **Figure S2.** The ability of two lncRNAs based on the signature to predict prognosis for PC patients from the TCGA database. **Figure S3.** (A) Based on TCGA data, the two-lncRNA nomogram’s prediction capacity was assessed using Kaplan-Meier analysis, log-rank P, and C-index. The Kaplan-Meier curves depict and compare OS time between low- and high-risk groups. (B) The predictive ability of two-lncRNA nomograms based on ICGC data was assessed using Kaplan-Meier analysis, Log-rank P, and C-index. (C) The findings of the TCGA multivariate analysis showed that the 2-lncRNA signature might be used as a powerful predictor of PC OS rate compared to other clinicopathological variables. (D) The findings of the ICGC multivariate analysis revealed that 2-lncRNA features might be utilized to predict PC OS rate in the absence of other clinicopathological variables.

## Data Availability

The datasets used and/or analyzed during the current study are available from the corresponding author on reasonable request.

## Abbreviations

PC: Prostate cancer
MALAT1: Metastasis-associated lung adenocarcinoma transcripts 1
ncRNAs: Non-coding RNAs
PCR: Polymerase chain reaction
lncRNAs: Long non-coding RNAs
ORF: Open reading frame
NSCLC: Non-small cell lung carcinoma
GDC: Genomic Data Commons
FDR: False discovery rate
GO: Gene Ontology
KEGG: Kyoto Encyclopedia of Genes and Genomes
*RT-*PCR: Reverse transcription polymerase chain reaction
FITC: Annexin V-fluorescein isothiocyanate
PI: Propidium iodide
CTs: Threshold cycles
ECM: Extracellular matrix
CAMs: Cell adhesion molecules
CC: Cervical cancer
HCC: Hepatocellular carcinoma
Coef: Coefficient
