# Supplementary material for: Targeting the MALAT1 gene with the CRISPR/Cas9 technique in prostate cancer
Source: Genes Environ. 2022 Sep 26;44:22. doi: 10.1186/s41021-022-00252-3 (PMC9511773; doi:10.1186/s41021-022-00252-3)
Supplement: Supplementary file 4 — Additional file 4: Figure S1. (A) Prostate tumors and non-carcinoma tissue samples have different DElncRNAs. Differentially elevated lncRNAs are shown by red dots, down-regulated lncRNAs are represented by green dots, and black dots represent no genes. (B) The differentially expressed lncRNAs in prostate cancer and normal tissues were studied using unsupervised hierarchical clustering. (C) Multivariate Cox regression analysis generated a forest map of 2 lncRNAs. Figure S2. The ability of two lncRNAs based on the signature to predict prognosis for PC patients from the TCGA database. Figure S3. (A) Based on TCGA data, the two-lncRNA nomogram’s prediction capacity was assessed using Kaplan-Meier analysis, log-rank P, and C-index. The Kaplan-Meier curves depict and compare OS time between low- and high-risk groups. (B) The predictive ability of two-lncRNA nomograms based on ICGC data was assessed using Kaplan-Meier analysis, Log-rank P, and C-index. (C) The findings of the TCGA multivariate analysis showed that the 2-lncRNA signature might be used as a powerful predictor of PC OS rate compared to other clinicopathological variables. (D) The findings of the ICGC multivariate analysis revealed that 2-lncRNA features might be utilized to predict PC OS rate in the absence of other clinicopathological variables. [file 41021_2022_252_MOESM4_ESM.docx]

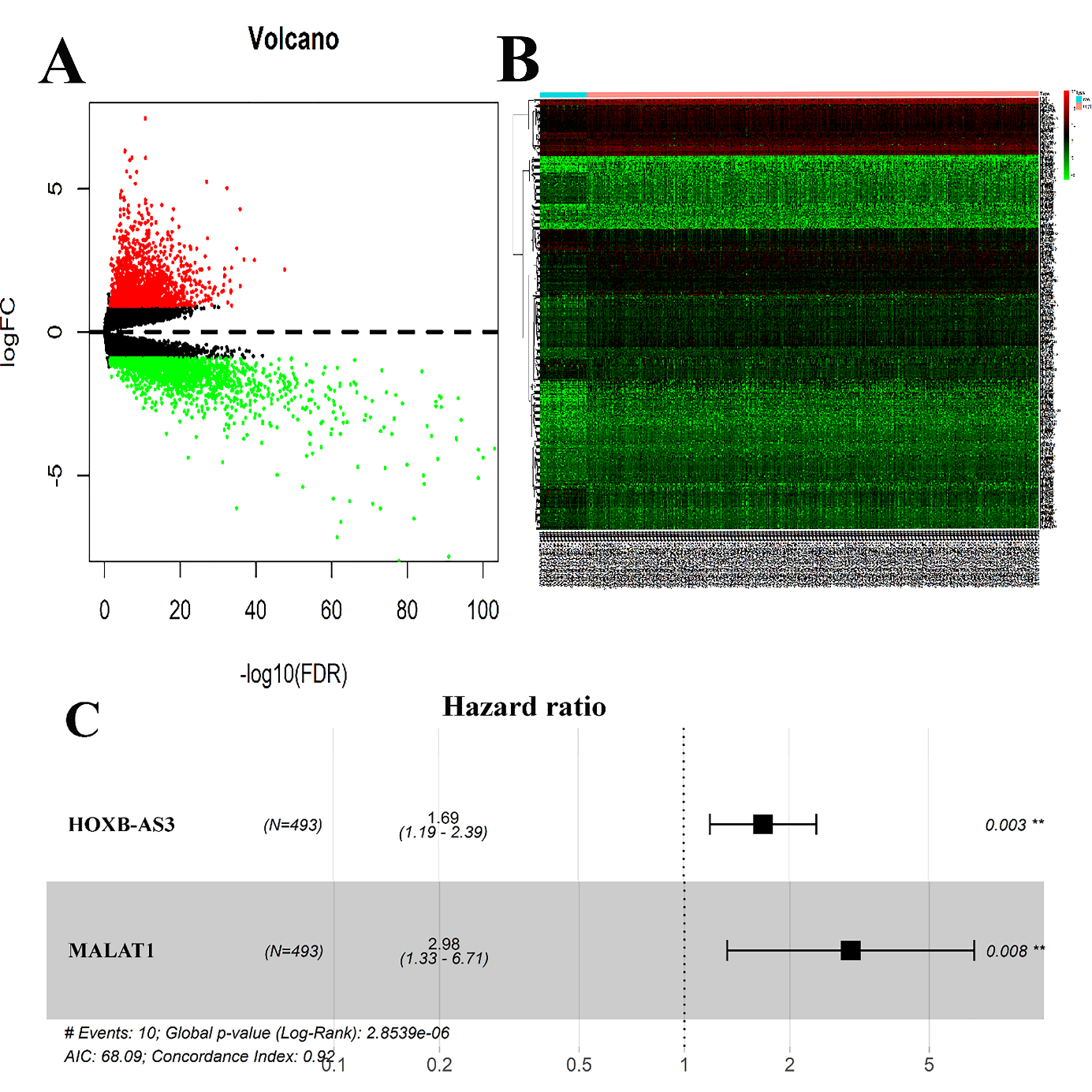


**Figure S1. (A)** Prostate tumors and non-carcinoma tissue samples have different DElncRNAs. Differentially elevated lncRNAs are shown by red dots, down-regulated lncRNAs are represented by green dots, and black dots represent no genes. **(B)** The differentially expressed lncRNAs in prostate cancer and normal tissues were studied using unsupervised hierarchical clustering. **(C)** Multivariate Cox regression analysis generated a forest map of 2 lncRNAs.


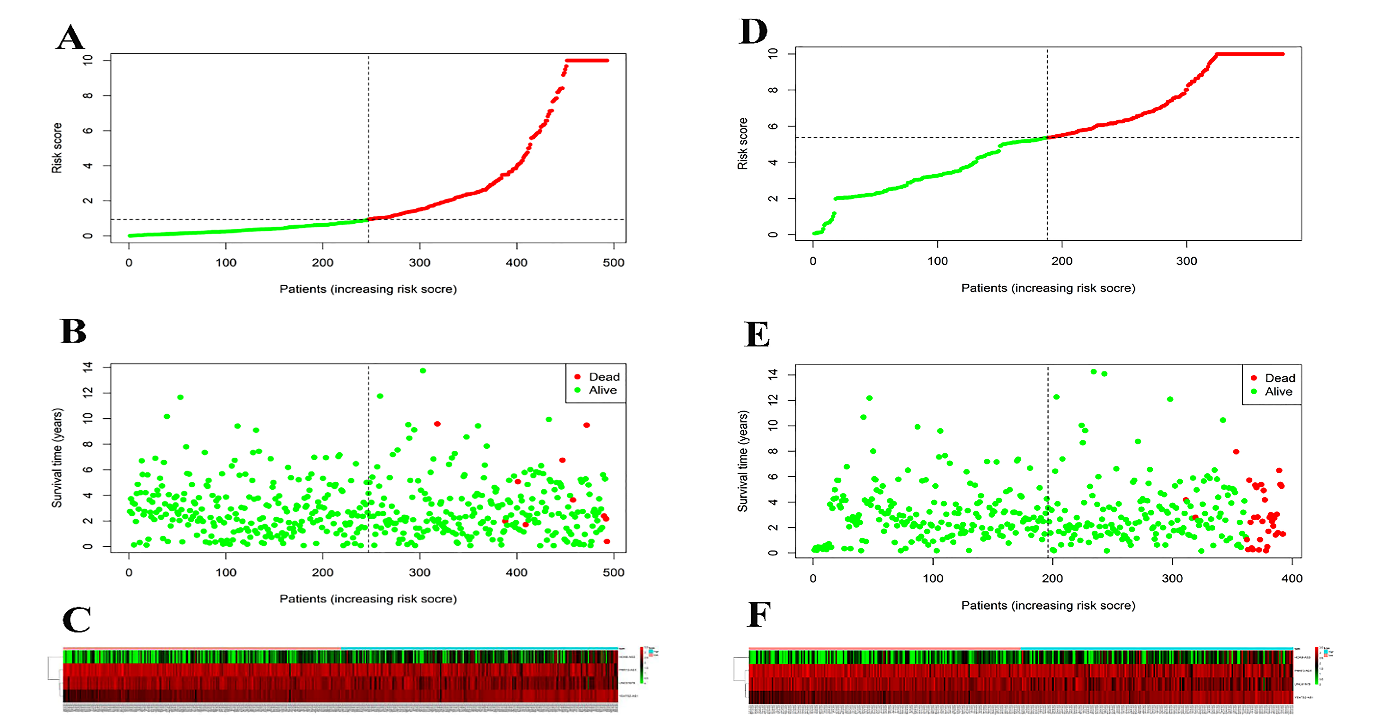


**Figure S2**. **The ability of two lncRNAs based on the signature to predict prognosis for PC patients from the TCGA database. (A)** Patients' risk scores are distributed. **(B)** The average length of time for a PC patient to survive. **(C)** A heatmap of the expression levels of the 2 lncRNAs used in the prognostic model. The vertical black dotted line represents the ideal threshold for dividing patients into high- and low-risk groups.

**The model's prognostic predictive power in patients with PC. (D)** Patient risk score distribution **(E)** PC patients' survival time. **(F)** Heatmaps of the prognostic model of two lncRNAs. The vertical dotted black line represents the appropriate threshold for categorizing instances into high- and low-risk categories.


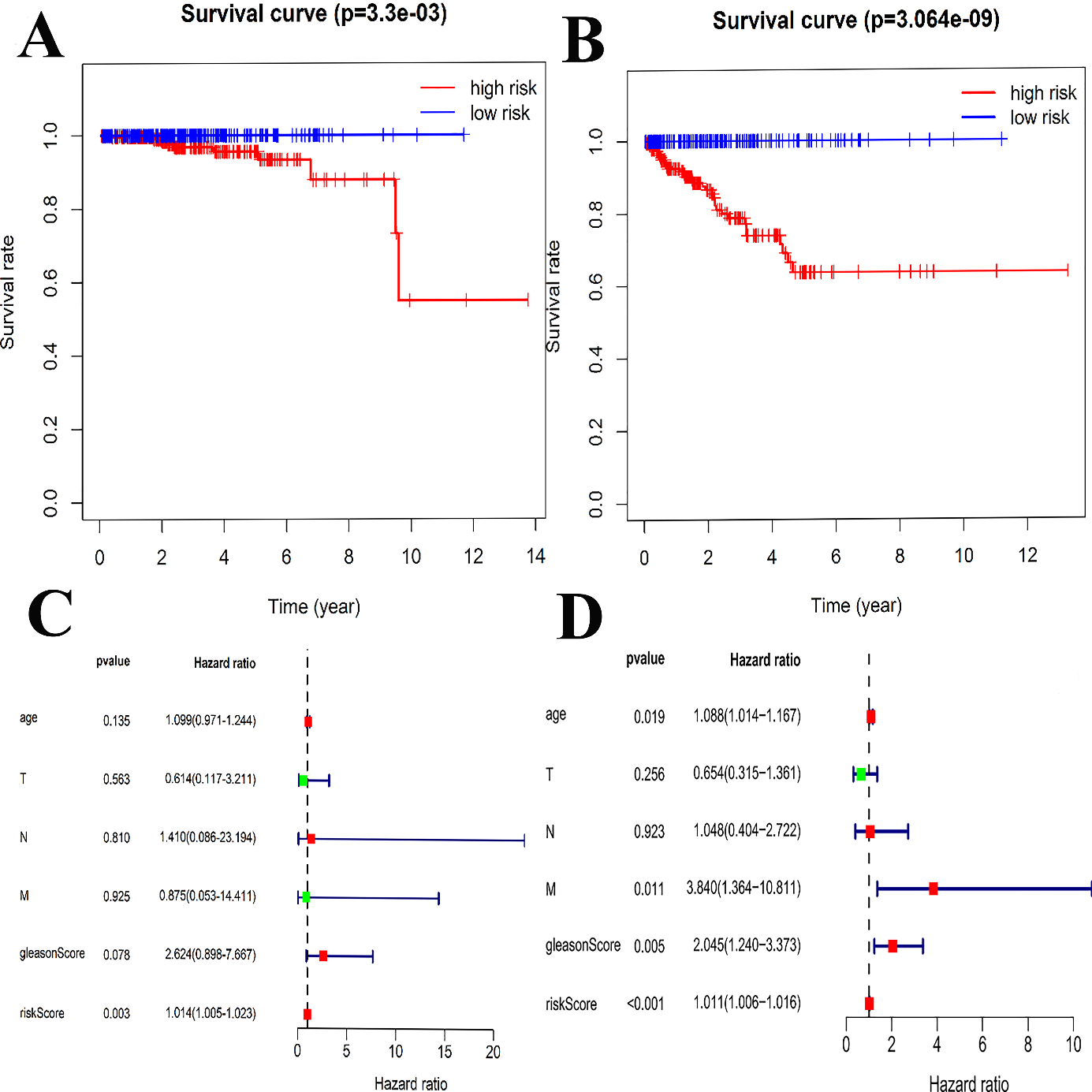


**Figure S3. (A)** Based on TCGA data, the two-lncRNA nomogram's prediction capacity was assessed using Kaplan-Meier analysis, log-rank P, and C-index. The Kaplan-Meier curves depict and compare OS time between low- and high-risk groups. **(B)** The predictive ability of two-lncRNA nomograms based on ICGC data was assessed using Kaplan-Meier analysis, Log-rank P, and C-index. **(C)** The findings of the TCGA multivariate analysis showed that the 2-lncRNA signature might be used as a powerful predictor of PC OS rate compared to other clinicopathological variables. **(D)** The findings of the ICGC multivariate analysis revealed that 2-lncRNA features might be utilized to predict PC OS rate in the absence of other clinicopathological variables.
